# Efficacy of concentrated growth factors combined with mineralized collagen on quality of life and bone reconstruction of guided bone regeneration

**DOI:** 10.1093/rb/rbaa007

**Published:** 2020-04-06

**Authors:** Yan Dai, Xiao-Hui Han, Li-Hua Hu, Hai-Wei Wu, Sheng-Yun Huang, Yu-Peng Lü

**Affiliations:** r1 Department of Oral and Maxillofacial Surgery, Central Hospital of Zibo, Shandong University, Zibo 255036, China; r2 Department of Oral and Maxillofacial Surgery; r3 Department of Prosthodontics, Shandong Provincial Hospital Affiliated to Shandong University, Jinan 250021, China; r4 Key Laboratory for Liquid-Solid Structural Evolution and Processing of Materials, Ministry of Education, Shandong University, Jinan 250061, China; r5 School of Materials Science and Engineering, Shandong University, Jinan 250061, China

**Keywords:** concentrated growth factors, mineralized collagen, guided bone regeneration, bone augmentation, osseointegration

## Abstract

To evaluate the clinical efficacy of concentrated growth factors (CGFs) combined with mineralized collagen (MC) in guided bone regeneration (GBR). A retrospective study involving 29 patients treated with GBR technique, which was performed either CGF and MC complexes or MC alone. Implants were inserted simultaneously and cone-beam computed tomography was taken immediately, at 3 and 6 months postoperation. Questionnaires were completed by all patients so as to evaluate the main symptoms and daily activities during the first week after surgery. The outcomes of the two groups were statistically compared. All implants healed uneventfully. Patients in both groups suffered from different levels of discomfort for the reason of swelling, pain and chewing impairment on 1–2 days. Meanwhile, swelling of the Trial group was weaker than the Control group. When compared with the Control group, pain levels in Trial group were more rapidly reduced and patients took fewer analgesics from Day 3. Furthermore, the reconstitution mean value of the graft was thicker at 3 and 6 months in Trial group. CGFs complex with MC were beneficial to relieve the clinical symptoms, promote the peri-implant bone regeneration and shorten the healing time.

## Introduction

Alveolar ridge resorption has been considered as an inevitable consequence of tooth extraction for a long time [1, 2]. The deficiency of alveolar bone presents a clinical problem for implant placement. The width of residual bone on buccal and lingual aspects must be at least 1 mm in order to maintain crestal bone levels, which means bone augmentation procedures should be performed when the width of alveolar ridge is <5-mm wide [3]. Hence, guided bone regeneration (GBR) technique, using barrier membranes and bone substitutes such as autografts, allografts, xenografts and alloplasts, has been applied for the reconstruction of defect region [4]. Autografts taken from an adjacent or remote site in the same patient is regarded as the ‘gold standard’ [5]. Due to the shortcomings of limited donor bone grafts, demanding for second surgical procedure and unpredictable resorption, it is imperative to explore alternatives to autografts [6, 7]. Bone substitutes may be applied to avoid these disadvantages without any volume limitations. Allografts such as demineralized freeze-dried bone allograft have properties of osteoconductive and osteoinductive [8]. Xenografts provide scaffolds for new bone regeneration and only possess osteoconductive properties [9]. However, allografts and xenografts have disadvantages of disease transmission, immune rejection and ethical issues.

The biomimetic mineralized collagen (MC), as newly alloplastic graft materials designed by Cui and colleagues [10], consists of orderly arranged nano-hydroxyapatite and Type I collagen. Furthermore, MC has good osteogenic activity, and its composition and microstructure are consistent with natural bone, which has been widely used for bone defect repair in clinic [11, 12].

CGF, first developed by Sacco in 2006, are new generation of platelet concentrated products and become the supplement of bone graft materials [13]. They are produced by centrifuging blood samples with a special centrifuge device. Differential centrifugation results in formatting more growth factors and more rigid fibrin structures than those observed in platelet-rich plasma (PRP) and platelet-rich fibrin (PRF). In addition, it has been sated recently that CGF tend to be more effective in bone regeneration or soft tissue healing [14]. Application of CGF could also significantly increase osteogenesis in sinus augmentation [15].

Research about the compound of MC and CGF applying as bone graft material in GBR has not been reported. We hypothesized that application of CGF and MC in GBR would improve treatment outcomes. Therefore, the purpose of this study was to assess postoperative complications such as pain, swelling and trismus in both groups. Meanwhile, we also wanted to evaluate dimensional changes in bone augmentation between groups.

## Materials and methods

### Study population and design

Hospital records from January 2016 through June 2018 were retrospectively assessed to identify patients who suffered from with bone deficiency and required GBR protocol. A total of 29 patients from Department of Oral and Maxillofacial Surgery of Shandong Provincial Hospital Affiliated to Shandong University were divided into two groups: the Trial group who were grafted with CGF plus MC (Allgens^®^, Beijing Allgens Medical Science and Technology Co., Ltd., China) and the Control group who were grafted with MC.

### Inclusion/exclusion criteria

The inclusion criteria were as follows:

- 18 years≤aged≤60 years
- No history of systemic disease that not suitable for oral surgery
- Periodontal condition with good plaque control
- residual bone possesses sufficient width of 2–4 and height >3mm

Exclusion criteria were as follows:

- Systemic disease that affect bone healing, such as uncontrolled diabetes, osteoporosis and HIV etc.
- Pregnancy and lactation
- Previous or current radiation or immunosuppressive therapy
- Smoking and excessive drinking

### Presurgical treatment

Clinical examination and cone-beam computed tomography (CBCT) were taken for each patient before the operation. Furthermore, all patients received periodontal treatment and oral hygiene instructions to provide a better oral environment.

Gargle with 0.2% chlorhexidine gluconate for 1 min. All patients received systemic antibiotics (Roxithromycin Capsules of 150 mg) prophylactically 1 h prior to surgery.

### CGF preparation

**Figure 1:**
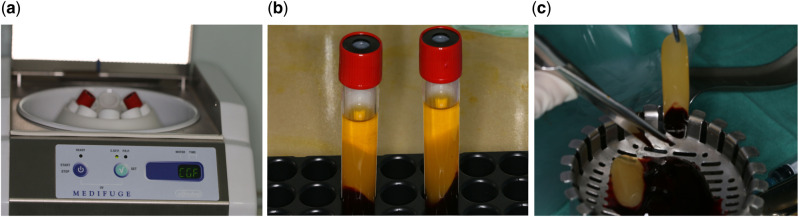
Preparation of CGF. (**a**) Blood centrifugation; (**b**) after centrifugation; (**c**) separate the CGF layer

Autologous CGF was prepared from fresh venous blood of patients. The venous blood samples were taken into 2 sterile 10 ml tubes without anticoagulants. The samples were immediately centrifuged with CGF centrifuge machine (Medifuge, Silfradent, Italy; Fig. 1a) with the following fixed procedures: 30″ acceleration, 2′ 2700 rpm, 4′ 2400 rpm, 4 2700 rpm, 3′ 3000 rpm, 36″ deceleration and stop. Centrifugation divided the blood into four layers: (i) red blood cell layer at the bottom (ii) CGF at the second layer (iii) the buffy coat at the third layer and (iv) the upper supernatant layer (Fig. 1b). The CGF layer was mechanically separated using sterile scissors (Fig. 1c). The CGF layer was then placed in a condensing disc and mixed with MC in 1:1 ratio (Allgens^®^, Beijing Allgens Medical Science and Technology Co., Ltd., China).

### Surgical procedure

All patients were operated by Dr S.-Y.H. The procedure was performed under local anesthesia (4% articaine with 1:100 000 epinephrine).

The Nobel implant system (NobelActive^®^/NobelReplace™, Nobel Biocare, Göteborg, Sweden), the XIVE implant system (XIVE^®^, Dentsply Friadent, Mannheim, Germany) and the DIO implant system (DIO-SM, Busan, South Korea) were used in this study.

Incisions were made from the one side of alveolar ridge crest to the other, with vertical incisions on either side. Then the periosteum was detached from the bone surface so as to expose both the labial and palatal/lingual aspects of the alveolar ridge. Based on the presurgical CBCT, implants with appropriate dimensions were placed using routine process. The final sitting of implant was achieved primary stability of 30 Ncm or more, and the cover screw was placed. Implant sits were treated for GBR. The membrane (Heal-All^®^ Oral Cavity Repair Membrane, Yantai Zhenghai Bio-tech Co., Ltd., China) was trimmed to a desirable dimension, and carefully covered the bone material. 3/0 Vicryl were used to sutures the flap closely (Vicryl Rapid-Ethicon Johnson, Diegem, Belgium). Then CBCT (ProMax 3D, Planmeca OY, 00880 Helsinki, Finland) scans were performed after surgery.

Patients were instructed to take an analgesic (LOXONIN^®^, 60 mg) after the surgical intervention and cold compresses also recommended. Postoperative prescriptions included antibiotics (Roxithromycin Capsules of 150 mg) for 3 days, 0.2% chlorhexidine oral rinse twice a day for 6 days (starting the day after surgery) and analgesic medication (LOXONIN^®^, 60 mg)if necessary. All patients were scheduled for recall at 7–10 days for suture removal.

**Figure 2:**
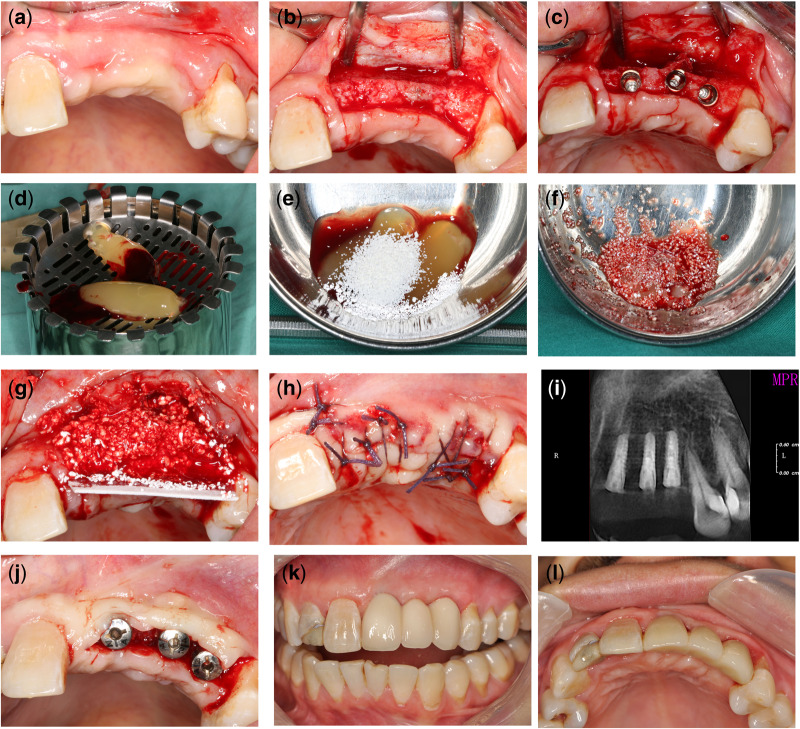
(**a**) Edentulous alveolar ridge in the front maxilla before surgery; (**b**) incisions from mesial of right maxillary Central incisor to the left maxillary premolars; (**c**) implant placement; (**d**) preparation of CGF; (**e**) the CGF and MC was mixed in 1:1 ratio; (**f**) complex of CGF and MC (**g**) GBR procedure; (**h**) interrupted suture; (**i**) radiograph after placement; (**j**) second stage surgery; (**k, l**) final zirconia crown

Second stage surgery was performed 3–4 months after implantation and healing abutment was inserted. Then reconstructive treatment protocol was initiated 2 weeks later (Fig. 2a–l).

### Clinical and radiological analysis

**Figure 3:**
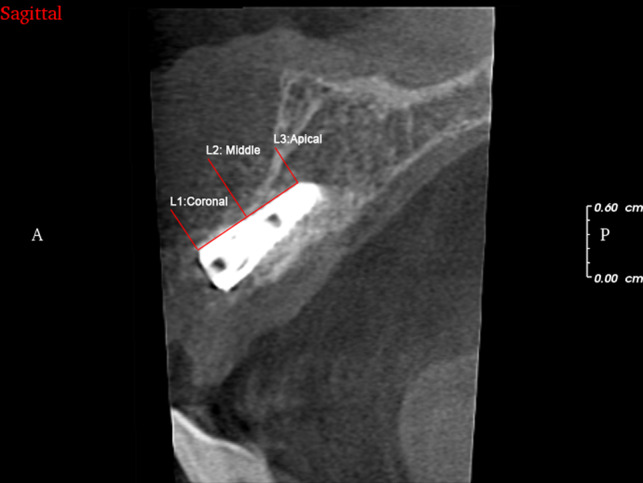
Method of measurement after GBR surgery

CBCT were taken immediately after operation, at 3 and 6 months postsurgery. Method of measurement was similar to that used in previous studies [16, 17]. First, the middle of implant diameter was confirmed in coronal position. Distance from the external surface of the labial bone to the buccal surface of the implant were measured at coronal level (L1), middle level of the implant (L2) and the implant apical (L3; Fig. 3). These parameters were measured at least three times, and the mean values were recorded.

A questionnaire followed by Tsesis *et al.* [18] was filled out by every patient starting the day of surgery for 7 days postoperatively and was applied to assess postoperative patients’ limitations (sleeping, mastication, interincisor distance, phonetics, daily routine and work, pain and other symptoms, such as bleeding, swelling, nausea and bad taste/breath). According to their daily life, patients should answer the 10 questions in the questionnaire with the 5-point Likert-type scale from 1 (‘not at all’) to 5 (‘very much’; Table 1).

**Table 1:** Quality of life questionnaire

	None	Little	Some	Quite a bit	Very much
Do you experience any difficulties with mouth opening?	1	2	3	4	5
Do you experience any difficulties with chewing?	1	2	3	4	5
Do you experience any difficulties with speaking?	1	2	3	4	5
Do you experience any difficulties with sleeping?	1	2	3	4	5
Have you missed your work/school?	1	2	3	4	5
Do you experience any difficulties with your daily activities?	1	2	3	4	5
Do you have swelling?	1	2	3	4	5
Do you have bleeding?	1	2	3	4	5
Do you feel nausea?	1	2	3	4	5
Do you feel a bad taste or breath?	1	2	3	4	5

Did you take any pain-killers today? ______.

Visual analog scales (VASs) scores appeared to be effective tools for assessing dental pain perception, using ‘0 = no pain’ and ‘100 = the most intense pain imaginable’ [19]. The final question involved whether the patient had taken any analgesics on each postoperative day. Questionnaires were returned in 7-day subsequent visit by patients.

### Statistical analysis

The two-sample t-test was performed to evaluate statistical differences between the two groups for new buccal plate bone. Fisher exact test was used to evaluate statistically the difference between the groups for analgesics taken as well as differences in any variable related to activities (sleeping, mastication, interincisor distance, phonetics, daily routine and work) and symptoms (bleeding, swelling, nausea and bad taste/breath) on each day after surgery. Patients’ experience of quality of life in pain by VAS scores was assessed using the Shapiro–Wilk test. Statistical significance was considered to be *P* ≤ 0.05. Statistical analysis was performed by SPSS (SPSS 22.0, SPSS Inc., Chicago, IL, USA).

## Results

In total, 29 patients did not have complications and achieved clinically osseointegrated. Although there was no histological analysis described the characteristics of the tissue contacting the implant, the soft tissues surrounding the implants appeared free of inflammation. Thus, there were 15 patients estimated in Control Group (7 male and 8 female, aging from 20 to 58 years and 18 implants) and 14 patients (6 male and 8 female, aging from 26 to 57 years and 17 implants) in Trial Group. There were no statistically significant differences in distribution of patients on the basis of age, gender, smoking history, implant brand and implant site between the two groups(*P* > 0 .05; Table 2).

**Table 2:** Demographic information of the Control/Trial group

Patient no.	Sex	Age	Smokers	Implant brand	Implant site	Implant dimension (mm)	Insertion torque (Ncm)
Control							
1	F	33	No	XIVE^®^	11 21	3.8 × 9.5	30
2	M	20	Light	NobelActive^®^	43	4.3 × 13	35
3	M	45	No	XIVE^®^	35	3.8× 8	30
4	F	58	Light	DIO-SM	37	4.1 × 8	35
5	M	43	No	NobelReplace^TM^	46	4.3 × 11.5	30
6	F	28	Light	DIO-SM	37	4.5 × 10	35
7	M	37	No	DIO-SM	11	3.8 × 10	40
8	F	52	No	XIVE^®^	14	3.8 × 11	30
9	F	45	Light	DIO-SM	24	3.8 × 12	30
10	M	24	No	NobelActive^®^	31 32 42	3.5 × 10	35
11	M	31	No	XIVE^®^	21	3.8 × 13	35
12	F	29	No	XIVE^®^	46	4.5 ×9.5	25
13	M	49	No	NobelReplace^TM^	37	4.3 × 11.5	30
14	F	31	No	XIVE^®^	12	3.4 × 11	30
15	M	38	No	NobelActive^®^	46	4.3 × 10	35
Trial							
1	M	29	No	DIO-SM	47	4.1 × 8	35
2	M	43	No	XIVE^®^	45	3.8 × 9.5	30
3	F	57	No	XIVE^®^	46	3.8 × 9.5	40
4	F	52	Light	NobelReplace^TM^	37	4.3 × 11.5	35
5	M	50	No	NobelActive^®^	11	3.5 × 11.5	30
6	F	28	Light	XIVE^®^	15	3.8 × 9.5	35
7	M	45	No	DIO-SM	46	4.5 × 10	25
8	F	26	No	XIVE^®^	12	3.8 × 13	30
9	F	40	No	DIO-SM	21 22 23	3.8 × 10	30
10	M	27	No	NobelActive^®^	13	3.5 × 11.5	35
11	F	31	No	XIVE^®^	36 37	4.5 × 9.5	35
12	F	36	Light	DIO-SM	25	3.8 × 10	30
13	M	30	No	NobelReplace^TM^	25	3.5× 11.5	30
14	F	54	No	XIVE^®^	21	3.4 × 11	30

### Clinical outcomes

All patients suffered from different levels of discomfort for the reason of swelling, pain and chewing impairment on the first day after surgery. The items of daily routine life and missed work were reported in either group, especially in the first 2 days. Whereas, these uncomfortable experiences were relieved more rapidly in Trial Group than that in Control group from the second day. The recovery of sleeping and phonetics was similar in both groups. When compared with the Trial group, swelling was more serious in Control group from Days 2 to 5.There was no difference in bleeding, nausea and bad taste/breath between the two groups (Tables 3 and 4).

**Table 3:** The outcomes of the evaluation for functional symptoms

	Day 1 (%)		Day 2 (%)		Day 3 (%)		Day 4 (%)		Day 5 (%)		Day 6 (%)		Day 7 (%)	
Symptom	C	T	C	T	C	T	C	T	C	T	C	T	C	T
Bleeding														
Very much														
Quite a bit														
Some	86.7	78.6	13.3	7.1										
Little/None	13.3	21.4	86.7	92.9	100	100	100	100	100	100	100	100	100	100
Swelling														
Very much	13.3	14.3	40.0	21.4	53.3		26.7							1
Quite a bit	53.3	57.1	40.0	28.6	33.3	21.4	40.0		26.7					
Some	33.3	28.6	20.0	50.0	6.7	50.0	20.0	28.6	33.3		13.3			
Little/none					6.7	28.6	13.3	71.4	40.0	100	86.7	100	100	100
Nausea														
Very much														
Quite a bit														
Some	20.0	7.1												
Little/none	80.0	92.9	100	100	100	100	100	100	100	100	100	100	100	100
Bad taste/breath														
Very much	6.7													
Quite a bit	20.0	21.4	6.7											
Some	40.0	35.7	40.0	42.9	20.0	14.3	6.7							
Little/none	33.3	42.9	53.3	57.1	80.0	85.7	93.3	100	100	100	100	100	100	100

**Table 4:** The outcomes of the evaluation for functional activity

	Day 1 (%)		Day 2 (%)		Day 3 (%)		Day 4 (%)		Day5 (%)		Day 6 (%)		Day 7 (%)	
Activity	C	T	C	T	C	T	C	T	C	T	C	T	C	T
Sleeping														
Very much	33.3	28.6	20.0	14.3	6.7									
Quite a bit	46.7	35.7	26.7	28.6	20.0	14.3								
Some	20.0	35.7	40.0	35.7	26.7	35.7	26.7	21.4	6.7					
Little/none			13.3	21.4	46.7	50.0	73.3	78.6	93.3	100	100	100	100	100
Mastication														
Very much	66.7	64.3	53.3	50.0	26.7	14.3	13.3	7.1	13.3	7.1				
Quite a bit	20.0	21.4	20.0	14.3	40.0	21.4	6.7	14.3	6.7	7.1	6.7			
Some	13.3	14.3	20.0	21.4	20.0	21.4	53.3	21.4	13.3	14.3	13.3			
Little/none			6.7	14.3	13.3	42.9	20.0	57.1	66.7	71.4	80.0	100	100	100
Interincisor distance														
Very much	53.3	50.0	33.3	35.7	26.7	7.1	13.3							
Quite a bit	20.0	28.6	20.0	21.4	20.0	7.1	6.7							
Some	26.7	21.4	20.0	14.3	13.3	14.3	13.3	7.1	13.3					
Little/None			26.7	28.6	40.0	71.4	66.7	92.9	86.7	100	100	100	100	100
Phonetics														
Very much		7.1												
Quite a bit	66.7	64.3	53.3	50.0	26.7	21.4								
Some	33.3	28.6	33.3	35.7	13.3	7.1	6.7							
Little/none			13.3	14.3	60.0	71.4	93.3	100	100	100	100	100	100	100
Daily routine														
Very much	6.7													
Quite a bit	26.7	28.6	20.0	14.3	6.7									
Some	53.3	35.7	26.7	21.4	13.3									
Little/none	13.3	35.7	53.3	64.3	80.0	100	100	100	100	100	100	100	100	100
Missed work														
Yes	86.7	92.9	80.0	85.7	66.7	50.0	20.0							
No	13.3	7.1	20.0	14.3	33.3	50.0	80.0	100	100	100	100	100	100	100

**Figure 4:**
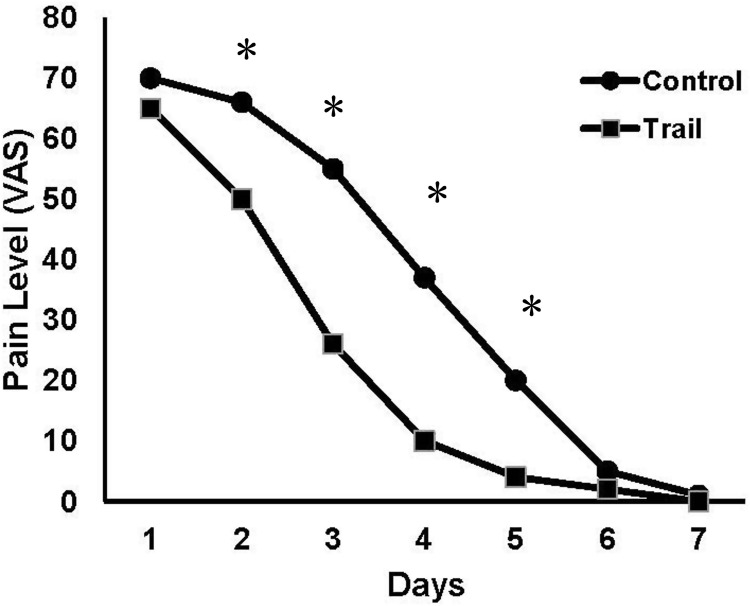
The levels of pain reported in the first week after surgery

**Figure 5:**
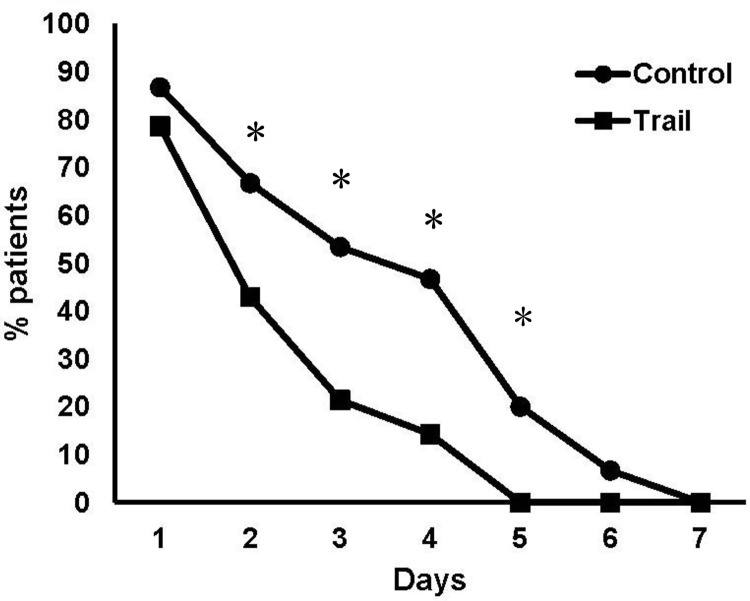
The proportion of patients taking analgesics in the first week after surgery

Furthermore, pain levels were rapidly reduced from Day 2 (Fig. 4) and patients took dramatically fewer analgesics on Day 2 in Trial Group compared with the Control group (Fig. 5; *P* < 0.05). The differences turned into negligible after Day 6.

### Radiographic outcomes

The radiographic evaluation confirmed that all the implants were healed uneventfully. The results (Table 5) indicated that the reconstitution mean value of the graft thickness was significantly decreased in the first 3 months. Moreover, the tendency was gradually stable between 3 and 6 months in both groups (*P* < 0.05). New bone formation in both groups was usually satisfactory. Importantly, the width of new buccal plate bone was thicker in Trial group at 3 and 6 months (*P* < 0.05).

**Table 5:** Distance from the external surface of the labial bone to the buccal surface of the implant

	Postsurgery		3 months		6 months	
	C	T	C	T	C	T
L1	2.50 ± 0.85	2.6 ± 0.55	2.13 ± 0.21	2.45 ± 0.57	2.09 ± 0.46	2.37 ± 0.77
L2	1.53 ± 0.49	1.49 ± 0.76	1.28 ± 0.70	1.39 ± 0.54	1.22 ± 0.42	1.35 ± 0.85
L3	1.48 ± 0.78	1.45 ± 0.31	1.30 ± 0.50	1.38 ± 0.32	1.25 ± 0.35	1.29 ± 0.36

## Discussion

In our study, CGF combined with MC achieved satisfactory effects in improving the quality of Life and bone reconstruction. To the best of our knowledge, this retrospective study first evaluated the clinical effects of CGF and MC as the grafting materials in GBR.

Platelet concentrated products such as PRP, plasma-rich in growth factors, PRF and concentrated growth factor (CGF) have been shown to be an efficient biomaterial for tissue regeneration [20]. As the third new generation of platelet concentrated products, CGF contains a variety of growth factors, such as platelet-derive growth factors (PDGFs), transforming growth factors β (TGFs-β), vascular endothelial growth factors (VEGFs), insulin-like growth factors, epidermal growth factor, fibroblast growth factor, as well as bone morphogenic protein. These factors can significantly promote healing of hard and soft tissue [13, 21]. Furthermore, a large number of CD34^+^ cells in CGF having been proved to play an important role in vascular maintenance, angiogenesis and neovascularization [13, 22]. Previous studies [23, 24] have demonstrated that CGF could validly accelerate the proliferation and differentiation of cells, promote wound healing processes and new bone formation. Reports also have indicated that CGF accelerated new bone formation in GBR and sinus grafting for many years [15, 25]. What’s more, CGF is widely applied for regeneration of alveolar ridge bone in combination with various biological materials or used alone [26, 27]. Ozveri Koyuncu *et al*. [28] reported that using CGF after third molar extraction significantly accelerated soft tissue healing and relieved the postoperative symptoms, particularly pain, swelling and trismus.

Based on the recognition of MC and its formation process, experts have focused on the preparation of biomimetic MC materials to imitate natural bone [29, 30]. MC is an artificial biomimetic with characteristics of osteogenic activity. Orderly arrangement of Type I collagen and nano-hydroxyapatite are the components of MC [10]. The Type I collagen was extracted from bovine tendon, and used as a template to form nano-sized hydroxyapatite through *in vitro* biomineralization [10, 12]. Feng *et al*. [11] reported that MC showed better effect on new bone formation in alveolar ridge preservation.

In our study, we assessed the effectiveness of CGF with MC on soft tissue healing and bone formation. As previously described in the Results section, patients in Trial group were tended to obtain more excellent quality of life after operation. With regard to soft tissue healing, our findings accorded closely with Ozveri Koyuncu *et al*.’s study [28], demonstrating that CGF is of great value for soft tissue healing and postoperative symptoms alleviation. The primary outcomes of our study showed that all patients suffered from discomforts such as swelling, pain and chewing impairment on the first day after surgery, while swelling was more serious in Control group from Days 2 to 5. When compared with the Control group, we found that the total amount of analgesic consumption in CGF group seemed to be lower than the Control group. Al-Hamed *et al*. [31] and Uyanık *et al*. [32] reported that fewer analgesic tablets were taken after PRF application, which was similar to our study.

CGF provides a powerful biological scaffold with and acts as an integrated reservoir to emit growth factors for accelerating tissue regeneration [22, 33–36]. What’s more, increased levels of transforming growth factor β-1 (TGF-β1), platelet-derived growth factor (PDGF), vascular endothelial growth factor (VEGF), interleukin-1β (IL-1β) and interleukin-6 (IL-6) in CGF contribute to promote soft tissue healing [37] and reduce postoperative complications. The secondary outcomes in the radiographic evaluation demonstrated that the reconstitution of new buccal plate bone in the Trial group was found to be more efficient than the Control group. Durmuslar *et al*. [26] evaluated CGF on the healing of peri-implant bone defects and restoration was achieved applied by autogenous bone and CGF. Wang *et al*. [27] found that Bio-Oss combined with CGF was more effective in increasing new bone formation than using Bio-Oss alone in a canine model. Honda *et al*. [38] implemented bone regeneration experiments on rat calvaria defects using CGF + BMSC and signaled remarkable healing of a critical-size bone defect *in vivo* in 2013. What’s more, Bonazza *et al*. [39] also showed that the combination of CGF and sodium orthosilicate stimulated cell proliferation and osteogenic differentiation, which could be effective in tissue regeneration. In agreement with the results of these previous studies, the application of CGF, our results showed better bone augmentation in Trial group with GBR protocol.

It is necessary to acknowledge some limitations in our study. One of the shortcomings was that the effect of CGF on bone regeneration was not evaluated. The other was that patients who participated in this study was limited to a short-term time-span and demanded for further observation to assess the success rate of implants.

## Conclusion

In conclusion, according to our results, application of CGF and MC has a positive impact on reducing postoperative discomforts and new bone formation. The complex of CGF and MC seems to be appropriate and efficient as a biomaterial for bone augmentation.

## Funding

This study was funded by China Postdoctoral Science Foundation Grant (No. 2019M652380). 

*Conflict of interest statement*. None declared.
